# Congress of multiple dimers is needed for cross-phosphorylation of IRE1α and its RNase activity

**DOI:** 10.26508/lsa.202302562

**Published:** 2024-06-17

**Authors:** Andrea Orsi, Eelco van Anken, Milena Vitale, Moreno Zamai, Valeria R Caiolfa, Roberto Sitia, Anush Bakunts

**Affiliations:** 1 Division of Genetics and Cell Biology, IRCCS Ospedale San Raffaele, Milan, Italy; 2 Division of Genetics and Cell Biology, Vita-Salute San Raffaele University , Milan, Italy; 3 Unit of Microscopy and Dynamic Imaging, Centro Nacional de Investigaciones Cardiovasculares (CNIC), Madrid, Spain; 4 Center for Experimental Imaging, IRCCS Ospedale San Raffaele, Milan, Italy

## Abstract

The unfolded protein response can switch from a pro-survival to a maladaptive, pro-apoptotic mode. During ER stress, IRE1α sensors dimerize, become phosphorylated, and activate XBP1 splicing, increasing folding capacity in the ER protein factory. The steps that turn on the IRE1α endonuclease activity against endogenous mRNAs during maladaptive ER stress are still unknown. Here, we show that although necessary, IRE1α dimerization is not sufficient to trigger phosphorylation. Random and/or guided collisions among IRE1α dimers are needed to elicit cross-phosphorylation and endonuclease activities. Thus, reaching a critical concentration of IRE1α dimers in the ER membrane is a key event. Formation of stable IRE1α clusters is not necessary for RNase activity. However, clustering could modulate the potency of the response, promoting interactions between dimers and decreasing the accessibility of phosphorylated IRE1α to phosphatases. The stepwise activation of IRE1α molecules and their low concentration at the steady state prevent excessive responses, unleashing full-blown IRE1 activity only upon intense stress conditions.

## Abstract (precis)

This work shows that dimerization is not sufficient to activate IRE1α and congregation of dimers is needed to trigger cross-phosphorylation and unleash endonuclease activity of this ER stress sensor.

## Introduction

Reliability of signal transduction is crucial for cell function and survival. The vast majority of secretory proteins fold and assemble in the endoplasmic reticulum (ER) under the assistance of resident chaperones and enzymes. Folding intermediates are retained in the early secretory compartment until they reach their native conformation (Anelli & Sitia, 2008). Proteins that fail to do so are cleared from the ER, most often through ER-associated degradation (ERAD), which entails dislocation to the cytosol for proteasomal degradation (Ellgaard & Helenius, 2003; Sitia & Braakman, 2003; Krshnan et al, 2022). When the load of clients overwhelms the folding capacity of the protein factory, ER stress ensues, which in turn activates three adaptive pathways (PERK, ATF6, and IRE1α) collectively referred to as the unfolded protein response (UPR) (Walter & Ron, 2011). The most conserved branch of the UPR is the one orchestrated by IRE1α. Upon ER stress, IRE1α is phosphorylated, oligomerizes, and acquires endonuclease activity, yielding spliced XBP1 mRNA (XBP1s). The resulting sXBP1 protein is a transcription factor that drives the expression of ER-resident chaperones, enzymes, and ERAD components (Walter & Ron, 2011). In such capacity, IRE1α plays a beneficial role as it is committed to re-establish ER homeostasis. However, under certain conditions, such as unresolved ER stress, IRE1α cleaves other RNAs in a process named regulated IRE1-dependent decay (RIDD; Hollien et al, 2009), which can cause apoptosis. Thus, a strict control of IRE1α activity is essential as inadequate regulation of the enzyme (both overactivation and underactivation) may lead to the premature death of otherwise healthy cells, or the survival of cells that instead ought to be eliminated. Its central role in cell life/death decisions is key in pathological processes such as cancer and diabetes, making IRE1α a promising therapeutic target (Morita et al, 2017; Raymundo et al, 2020).

Several reports have shown that upon intense stress, Ire1p forms clusters or foci, which have been proposed to help recruitment of HAC1 mRNA (yeast homolog of XBP1) or have a role in the acquisition of IRE1α RNase activity (Aragón et al, 2009; van Anken et al, 2014b). Inhibition of higher order oligomerization and attenuation of IRE1α RNase activity are mediated by BiP recruitment possibly via the J-domain of Sec63 (Li et al, 2020). However, much remains to understand on the chain of events that lead to and limit IRE1α activation. In this study, we analyzed a panel of mutants to dissect the stepwise role of dimerization, oligomerization, nucleotide binding, phosphorylation, and RNase activity during progression of ER stress.

We have found that surprisingly, IRE1α phosphorylation does not occur within isolated dimers, but only in trans upon collisions of dimers and/or formation of higher order oligomers. Owing to the low abundance of IRE1α, isolated dimers will bump into each other only occasionally—resulting in limited cross-phosphorylation. Thus, we infer that full-blown activation could be achieved only by formation of higher oligomers or by concentration of IRE1 dimers in specialized structures. The transition between dispersed, mildly activated IRE1 and fully active IRE1 in specialized foci may be crucial for controlling life/death cell decisions.

## Results

### IRE1α phosphorylation correlates in time and magnitude with its RNase activity

We recently developed and validated a robust cell model, which allows evaluation of ER homeostatic readjustments in response to a proteostatic insult, that is, the overexpression of orphan secretory Ig-µ heavy chain_s_ (μ_s_) (Bakunts et al, 2017; Vitale et al, 2019). Synthesis of exuberant levels of μ_S_ results in temporary shortage of free BiP, which leads to UPR activation. Using this model, we showed that reaching a new homeostatic equilibrium entails the transition from acute UPR signaling, when ER stress sensors are fully activated, to a chronic state characterized by an overall ER expansion (Bakunts et al, 2017; Vitale et al, 2019).

**Figure 1.**
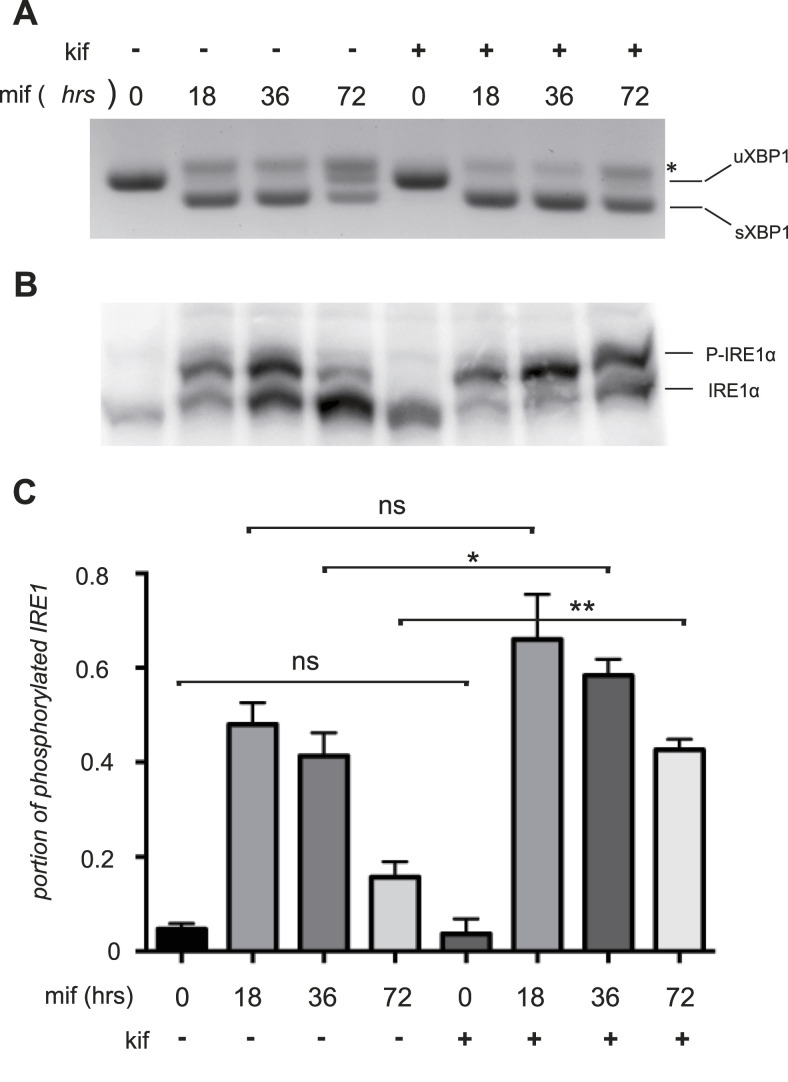
IRE1α phosphorylation level is proportional to its endonuclease activity. **(A)** HeLa-µs cells were induced with 0.5 nM mifepristone (Mif) to induce Ig-µ chain synthesis and treated with or without 30 μM kifunensine (kif) for the indicated times, to induce an adaptive or a maladaptive unfolded protein response, respectively. XBP1 splicing was used as an indicator of IRE1α endonuclease activity. A hybrid product that is formed during the PCR (Shang & Lehrman, 2004) is denoted by an asterisk. **(B)** Protein lysates from the same cells as in (A) were resolved by Phos-tag gels and blots decorated with anti-IRE1α. See panel (C) for densitometric quantifications. **(C)** Densitometric quantifications of the fraction of phosphorylated IRE1α. Preventing ER-associated degradation by kifunensine addition increased the extent of IRE1α phosphorylation, particularly during the late phases of the response to Ig-µ chain synthesis. A *t* test compared the portion of phosphorylated protein during Mif time course in the presence and the absence of kif (n = 3).

Here, we exploited our model to follow IRE1α phosphorylation at different stages of a proteostatically driven UPR. As previously described (Bakunts et al, 2017), at the early time-points, corresponding to an acute UPR, a significant portion of IRE1α is phosphorylated and high levels of spliced XBP1 are generated (Fig 1A and B). Later, when a new homeostatic equilibrium is established, IRE1α phosphorylation subsides to levels close to those in the steady state and its RNase activity decreases significantly. However, when ER stress cannot possibly be resolved—that is, because ERAD is blocked with the ERAD inhibitor kifunensine (kif)—the levels of IRE1α phosphorylation and XBP1 splicing remain high (Fig 1A–C) and cells eventually die. In this model, therefore, IRE1 phosphorylation and endonuclease activity parallel the intensity of stress.

### A cellular model to investigate IRE1 activation

**Figure 2.**
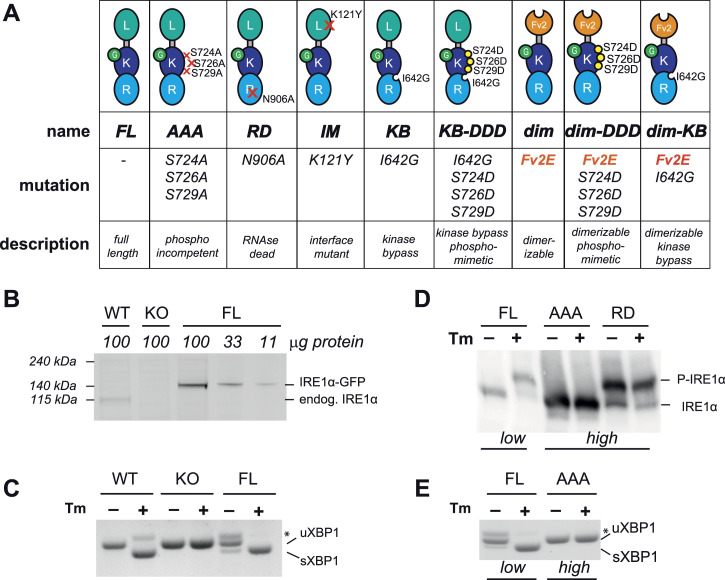
Phosphorylation precedes, and it is required for activation of IRE1 endonuclease. **(A)** Panel of all IRE1α mutants used in the study: L = luminal domain; K = kinase domain; R = RNase domain; G = mEGFP; Fv2E = artificially dimerizable domain. **(B)** Protein lysates from cells expressing endogenous IRE1α (WT), IRE1α-ablated cells (KO), and cells expressing inducible full-length IRE1α-GFP (FL) at minimal expression levels were resolved by SDS–PAGE and the blots decorated with anti-IRE1α. Different amounts of protein were loaded per lane, as indicated, to help compare the expression levels. Levels of FL-IRE1 at the lowest expression levels were estimated to be around fivefold those of endogenous IRE1, from this and other two independent experiments. **(C)** Activity of IRE1α in the cells described above was assessed by XBP1 splicing. IRE1 KO cells do not respond to Tm, whereas reconstituted cells expressing FL-IRE1 at low levels achieve robust XBP1 splicing. **(D)** Cells expressing low levels of full-length IRE1α (FL), and cells expressing high levels of phospho-incompetent (AAA) and RNase-dead (RD) mutants were treated with or without tunicamycin (Tm). Lysates were resolved by Phos-tag gels and blots decorated with anti-IRE1α. Although AAA-IRE1 cannot be phosphorylated in response to Tm, the RD-IRE1 mutant at the high expression level is phosphorylated even in the absence of ER stress. **(E)** Phosphorylation-deficient mutant (AAA) has no endonuclease activity even at high expression levels as assessed by the absence of sXBP1 after treatment with Tm.

**Figure S1.**
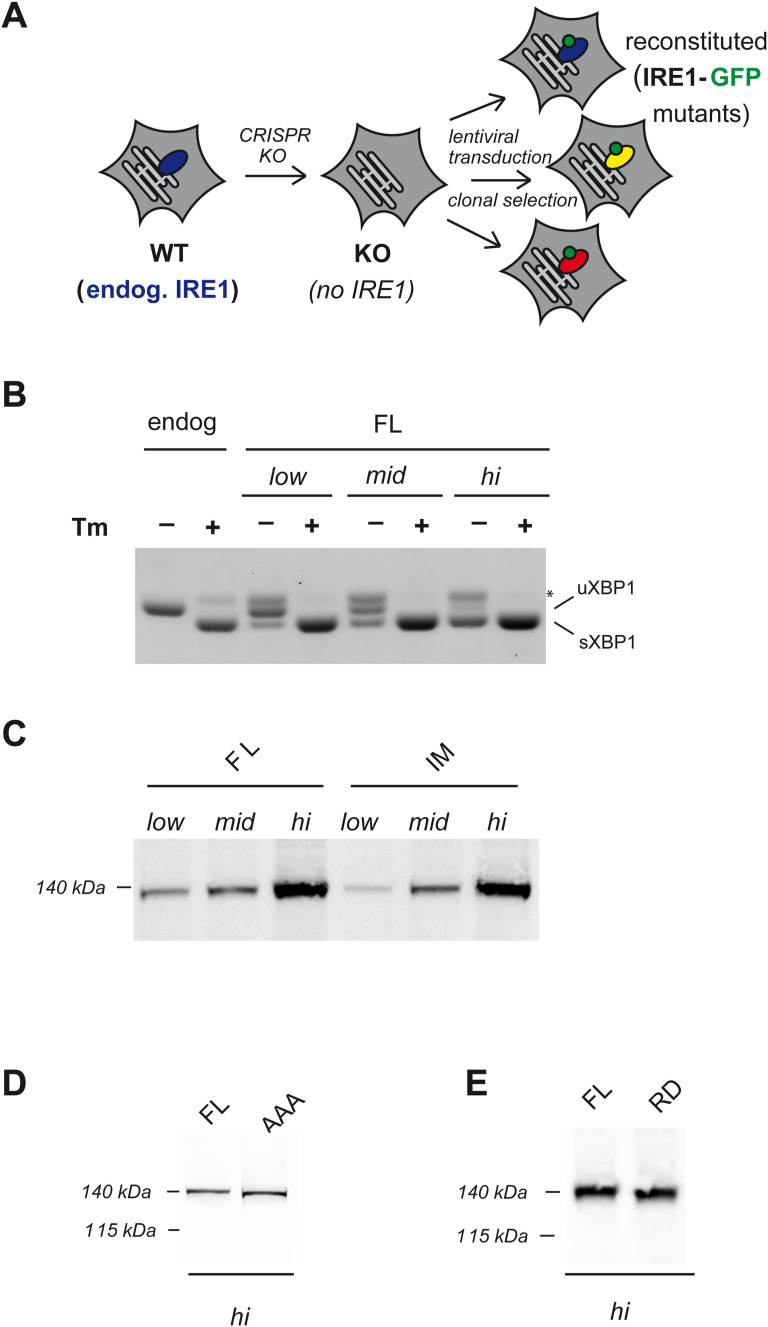
Generation of cell lines and expression levels of full-length IRE1 mutants. **(A)** Generation of HeLa cell lines used in the study. First, we generated IRE1 KO cells by CRISPR. Then, we reconstituted these cells by lentiviral transduction, with different Tet-inducible IRE1 mutants (see Fig 2A). For each mutant, we selected and characterized a clonal cell line, which showed consistent tunable expression. **(B)** Reconstituted cells expressing FL-IRE1 at different levels were treated with or without Tm. Their endonuclease activity was evaluated by XBP1 splicing and compared with that of wt cells expressing endogenous IRE1. Low levels of FL-IRE1 fully reconstitute XBP1 splicing. **(C, D, E)** Clonal HeLa cell lines expressing IRE1α mutants under the TetON-inducible promoter were treated with different concentrations of doxycycline to adjust the expression level of the transgenes. Aliquots from the cell lysates were resolved electrophoretically and the blots decorated with anti-IRE1α. Comparison between the expression levels of FL-IRE1 and the interface mutant (IM) of IRE1 (panel (C)); phospho-incompetent mutant (AAA) IRE1 (panel (D)); RNase-dead mutant (RD)-IRE1 (panel (E)).

To dissect the molecular steps that lead to IRE1α activation, we generated CRISPR-knockout cells for IRE1α and reconstituted them with a panel of IRE1α mutants designed to pinpoint the roles and relationships between dimerization, phosphorylation, nucleotide binding, and full enzymatic activation (Figs 2A and S1A). All our mutants were also tagged with mEGFP to enable imaging studies. For the sake of simplicity, we will refer to these IRE1α variants using the names in the table of Fig 2A, omitting the mEGFP tag.

In order to compare the activity of the mutants, it was essential to control their expression. To this end, we employed the TetON system and created inducible HeLa cell lines to express the different IRE1α mutants in a tunable way. With this inducible promoter, the lowest expression levels that we could achieve for full-length (FL) wild-type IRE1α (FL-IRE1α) were around five times that of endogenous IRE1α (Fig 2B). Therefore, we tuned the expression of each IRE1α mutant, at three comparable levels between each other: low, intermediate, and high (see Figs S1C–E and S4A).

At the low expression of FL-IRE1α, we detected minimal XBP1 splicing even in basal conditions (Figs 2C and S1B). However, upon treatment with tunicamycin (Tm), an inhibitor of N-glycosylation known to cause robust ER stress, FL-IRE1α responded promptly and restored efficient XBP1 splicing, showing that the protein can complement IRE1α KO cells as it is functional and responsive to stress.

**Figure 3.**
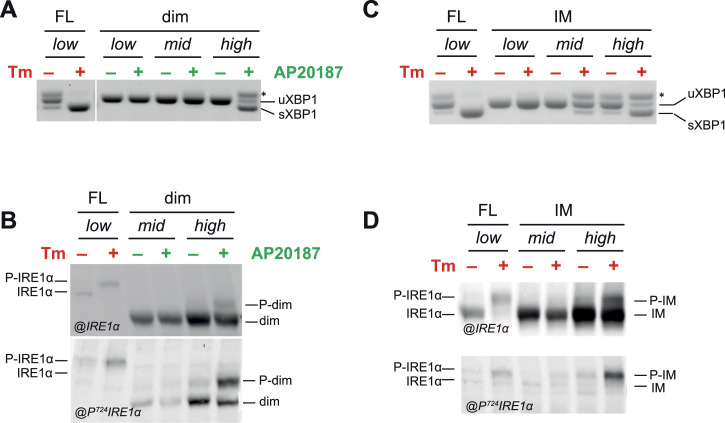
Dimerization is not sufficient for full-blown activation of IRE1α. **(A)** Endonuclease activity of dimerizable IRE1 (dim) upon treatment with dimerizing drug AP20187 (green). Dim-IRE1 is expressed at different expression levels, as indicated, and its activity is compared with FL-IRE1 (low) treated with or without Tm (red). Dim-IRE1 can splice XBP1 mRNA only at very high concentrations and in the presence of AP20187. **(B)** Protein lysates from cells treated with Tm (red) or AP20187 (green) as indicated were resolved on Phos-tag gels and immunoblotted with anti-IRE1α (upper panel) and anti-phosphoIRE1α (phospho-S724, lower panel) antibodies. The first two lanes contain lysates from cells expressing FL-IRE1 at low levels. In the remaining lanes are shown lysates from cells expressing dimerizable IRE1 (dim) at medium and high expression levels, respectively. Dimerizable IRE1α is partially phosphorylated only at high expression levels. P-dim, phosphorylated dim-IRE1. **(C, D)** Same as in panels (A, B) but for cells expressing the interface mutant (IM) of IRE1α treated or not with Tm for 4 h. Similar to dim-IRE1, IM-IRE1 can splice XBP1 and it is partially phosphorylated only at high expression levels. P-IM, phosphorylated IM-IRE1.

We then employed Phos-tag SDS–PAGE to monitor the phosphorylation status of FL-IRE1α. As confirmed by the mobility shift observed upon treatment with Tm, FL-IRE1α was efficiently phosphorylated (Fig 2D). The identity of IRE1 phosphorylated forms was further confirmed by specific anti-phosphoIRE1α antibodies (Fig 3B).

The importance of IRE1α phosphorylation as a prerequisite for endonuclease activity was confirmed by analysis of a full-length phosphorylation-deficient IRE1α mutant in which serines 724/726/729, located in the kinase activation loop of the protein, had been mutated to alanines (AAA-IRE1α). As expected, this mutant cannot be phosphorylated upon ER stress (Fig 2D). Differently from previous reports (Prischi et al, 2014), in our hands the AAA mutant was unable to splice XBP1 mRNA in response to ER stress. Endonuclease activity was not rescued even if the mutant was expressed at very high levels (Figs 2E and S1D).

Conversely, IRE1α carrying the N906A mutation, which abolishes its RNase activity (RNase dead, RD-IRE1α) (Han et al, 2009), is phosphorylated even in the absence of ER stress (Fig 2D), confirming that phosphorylation precedes and can occur independently from the endonuclease activity of IRE1α.

### IRE1α dimers are not capable of autophosphorylation

Dimerization is known to be a prerequisite for IRE1α activation. In principle, one IRE1α dimer could be sufficient for a single XBP1 cleavage event (Korennykh et al, 2011). However, it is not clear whether phosphorylation and the following steps require higher order IRE1α oligomerization. Indeed, in several cellular models ranging from yeast to mammals, IRE1α was shown to form oligomers and big signaling clusters upon acute ER stress (Aragón et al, 2009; Korennykh & Walter, 2012).

Tracking and controlling the transition between monomeric, dimeric, and oligomeric forms of IRE1α in a living cell is quite challenging. To tackle this problem, we created a dimerizable IRE1α chimera (dim-IRE1α) in which the luminal domain of IRE1α was replaced by a modified version of the FVBK domain (Fv2E). Two of such domains can be brought together artificially by the addition of the divalent chemical AP20187, as it was done before for another ER stress sensor, PERK (Lu et al, 2004; Lin et al, 2009). Thanks to this chimeric construct, the transition between monomeric and dimeric IRE1α can be manipulated in a tightly controlled fashion, independently from ER stress and without concomitant activation of other UPR sensors. Moreover, the dim-IRE1α mutant allows to assess the neat contribution of dimerization to phosphorylation and endonuclease activity, because this chimera lacks structural elements of the luminal domain that could mediate formation of stable IRE1α oligomers.

**Figure S2.**
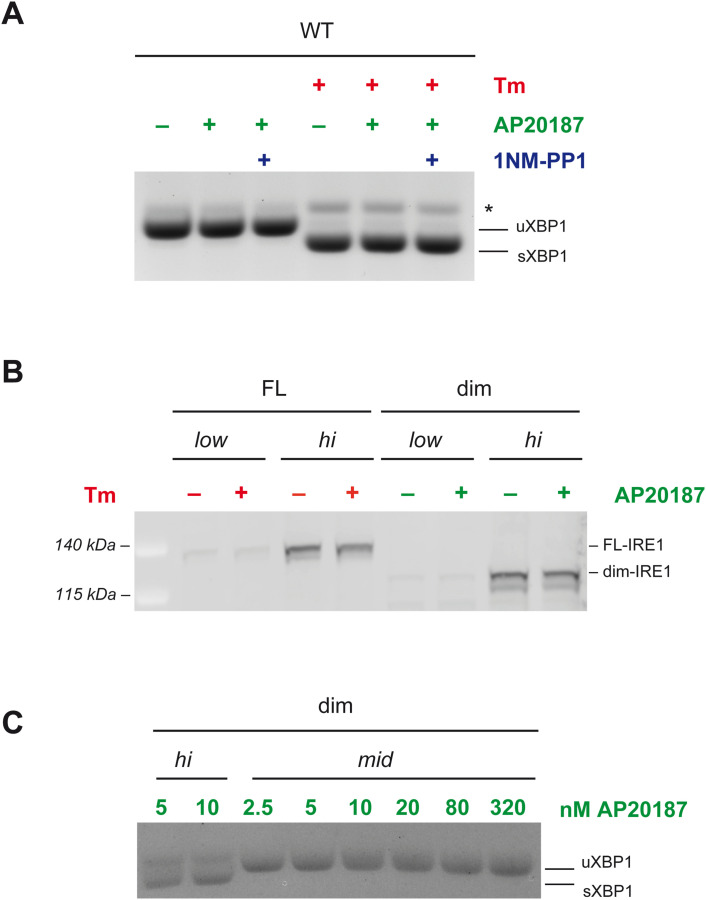
Treatment with drugs do not alter expression levels of IRE1 mutants, and AP20187 concentration is saturating. **(A)** Cells expressing endogenous IRE1 were treated with dimerizing drug AP20187, nucleotide analog 1NM-PP1, and/or Tm, as indicated. As shown by the XBP1 splicing assay, in none of the conditions AP20187 and/or 1NM-PP1 affected endonuclease activity of wt IRE1. **(B)** HeLa cells reconstituted with full-length (FL) or dimerizable (dim) IRE1 were treated with doxycycline to achieve low or high expression levels of the respective protein. Cells were then treated with Tm (red) or AP20187 (green). Neither of the treatments alter the expression levels of IRE1, as shown by immunoblot with anti-IRE1 antibodies. A similar result for two other IRE1 mutants is shown in Fig S4B. **(C)** Cells expressing dim-IRE1 at high or medium levels were treated with increasing concentrations of AP20187 as indicated. As shown by the XBP1 splicing assay, a concentration of 5 nM is already sufficient to activate endonuclease activity if dim-IRE1 is expressed at high levels. On the contrary, if dim-IRE1 is present in lesser amounts (medium expression) AP20187 is unable to trigger XBP1 splicing even at 320 nM concentration. Key for activation is therefore the concentration of IRE1 dimers. In the rest of the study, unless differently stated, we have used AP20187 at a concentration of 10 nM.

As expected, treatment with AP20187 had no effect in cells expressing FL-IRE1α: neither it induced XBP1 splicing per se, nor it altered the response to Tm in these cells (Fig S2A). Moreover, AP20187 did not alter in any way the expression levels of all the IRE1 mutants we tested (Figs S2B and S4B). On the contrary, in the absence of the dimerizing agent, the expression of dim-IRE1α did not result in XBP1 splicing. This held true even when dim-IRE1α was expressed at very high levels, showing that appending the Fv2E domain does not mediate IRE1α activation per se (Fig 3A).

If dimerization of IRE1α were enough to activate the protein, the treatment with AP20187 would result in phosphorylation and then activation of endonuclease activity of dim-IRE1. However, neither of those happened when the mutant was expressed at relatively moderate levels (Fig 3A and B, low and mid-expression). Only when dim-IRE1 was heavily overexpressed (about 12 times than FL-IRE1 at low levels), did it become phosphorylated—although to a very limited extent, and it was able to splice XBP1 (Fig 3A and B, high).

We also reasoned that the presence of the Fv2E domain might impair IRE1α kinase activity. To exclude this possibility, we generated a cell line expressing the interface mutant (IM) of IRE1α, which has an intact luminal domain but a single point mutation (K121Y) that impairs oligomerization propensity (Li et al, 2010; Sundaram et al, 2018). In our hands, at low expression levels, neither this mutant became phosphorylated in the presence of ER stress (Tm treatment), nor did it splice XBP1. However, similar to what we observed for dim-IRE1, when overexpressed, a small amount of IM-IRE1 did display phosphorylation and endonuclease activity (Fig 3C and D, mid- and high expression).

Both our approaches, using dim- and IM-IRE1, indicate that isolated dimers are not able to autophosphorylate themselves, at least in a stable manner. Along the same line, and using a different assay based on single-molecule tracking, Belyy et al also reported that at the steady state, IRE1α forms constitutive inactive homodimers that are not capable of self-phosphorylation (Belyy et al, 2022).

### IRE1α dimers can phosphorylate other dimers in trans

All our findings suggest that IRE1 phosphorylation is triggered by formation of stable oligomers or upon collisions between dimers: both events would be more frequent the higher the concentration of IRE1α. Considering its overexpression, the proportion of phosphorylated Fv2E-induced dimers was rather small. Yet, such an amount was comparable to that of FL-IRE1α fully phosphorylated upon ER stress (see Fig 3B, compare lane 2 with lane 6). Again, the level of phosphorylated IRE1α correlated with RNase activity, irrespective of the nature of the luminal domain. The same was true for the interface mutant that did not form oligomers efficiently (Sundaram et al, 2018) and displayed inefficient phosphorylation at moderate expression levels (Fig 3C and D).

Formation of IRE1α clusters upon induction of ER stress has been observed in several studies (Aragón et al, 2009; Li et al, 2010; van Anken et al, 2014b), and it would be an ideal way to increase the local concentration of IRE1 and thus promote its activation.

**Figure 4.**
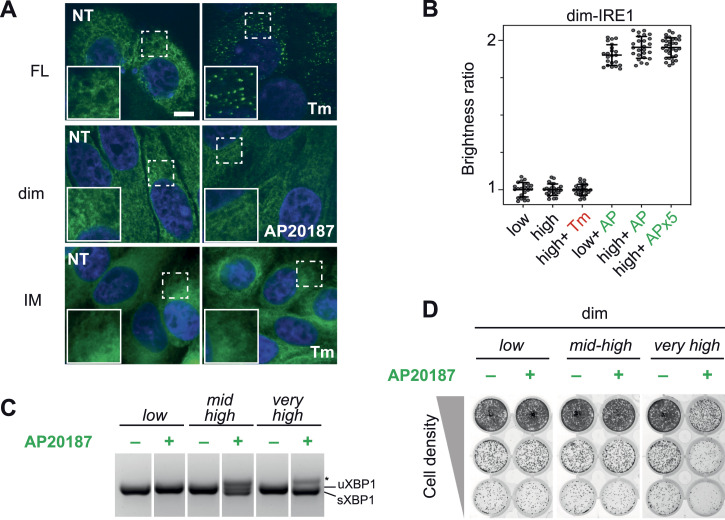
Congregation of IRE1α dimers achieves phosphorylation and activation of endonuclease activity. **(A)** Confocal images of cells expressing GFP-tagged FL-IRE1, dim-IRE1, and IM-IRE1. Neither dim-IRE1 nor IM-IRE1 form distinct foci upon treatment with Tm or AP20187, respectively, despite being expressed at high levels. All images have been acquired with the same magnification; scale bar (valid for all panels) = 10 μm. **(B)** Dimerization of dim-IRE1 in HeLa cells upon the addition of AP20187 determined by N&B analysis. The assembling of dim-IRE1 after treatment with or without AP20187 or Tm was assessed by analyzing the change in the fluorescence brightness of its mEGFP component (see the Materials and Methods section and Fig S3). dim-IRE1 was expressed at low and high concentrations, as indicated. In the absence of AP20187, only monomers of dim-IRE1 were detected at both low (n = 20 cells) and high (n = 23 cells) expression levels. Monomers were also prevalent after treatment with tunicamycin (n = 23 cells). Dimers were detected after treatment with 10 nM AP20187 at both expression levels (low: n = 22 cells; and high: n = 25 cells). Treatment with saturating fivefold higher concentrations of AP20187 (50 nM; APx5) did not result in the appearance of higher oligomeric forms of dim-IRE1 (n = 25 cells). The ratio 2:1 was obtained by dividing the average value of brightness (ε_i_) for each cell after treatment by the average value of brightness (ε_o_) for each control cell. Time stack images were collected after 2 h of incubation with the drugs. **(C)** Activity of dim-IRE1α at different expression levels in the presence and absence of AP20187 was assessed by XBP1 splicing. Note that the expression levels in this experiment were adjusted to deplete uXBP1 (very high) or have a very high level of XBP1 splicing but without depletion (medium–high). **(D)** Vitality of cells expressing dim-IRE1 at corresponding (see panel (C)) expression levels was evaluated by the crystal violet assay. Cells plated at different dilutions were induced with different amounts of doxycycline to induce expression as indicated, and then incubated with or without AP20187 for the rest of the experiment. Cells were fixed and stained with crystal violet after 7 d. Inducing the dimerization of IRE1 with AP20187 has no effect on cell vitality when dim-IRE1 is present in lesser amounts and displays no endonuclease activity (see below). The expression of dim-IRE at high levels is not toxic per se, but it becomes so upon depletion of uXBP1.

Because of the lack of the IRE1 luminal domain, the dim-IRE1α mutant cannot form foci (Fig 4A). The same was true for IM-IRE1, as the K121Y mutation prevented IRE1α from clustering in response to ER stress (Li et al, 2010 and Fig 4A). Still, both mutants were able, albeit in minimal part, to become phosphorylated and RNase-competent when present in large amounts, even in the absence of distinct foci. We reasoned that for these mutants, the high concentration bypasses the need for foci, as it makes interactions between dimers more likely.

Accordingly, Belyy et al (2022) reported that formation of IRE1 foci may not be required for phosphorylation and activation, but rather it ensues through interaction between inactive dimers. Both the Fv2E- and IM-IRE1 mutants that we used are supposedly impeded in oligomerization propensity (Sundaram et al, 2018; see below).

In our hands, therefore IRE1 activation seems to occur between dimers even in the absence of stable oligomers, provided that dimers are concentrated and their chance to bump into each other is high enough. To test this hypothesis, we undertook two different approaches.

First, we tested whether the activation of dim-IRE1 could be due to AP20187 promoting the formation of oligomers, for example, linking more dimers in trans. If that were true, increasing the amount of AP20187 should result in greater endonuclease activity of dim-IRE1. As shown in Fig S2C, this was not the case as the amount of AP20187 we used was sufficient to saturate the binding to the Fv2E domain, and much higher concentrations showed no difference in dim-IRE1 activation (Fig S2C).

**Figure S3.**
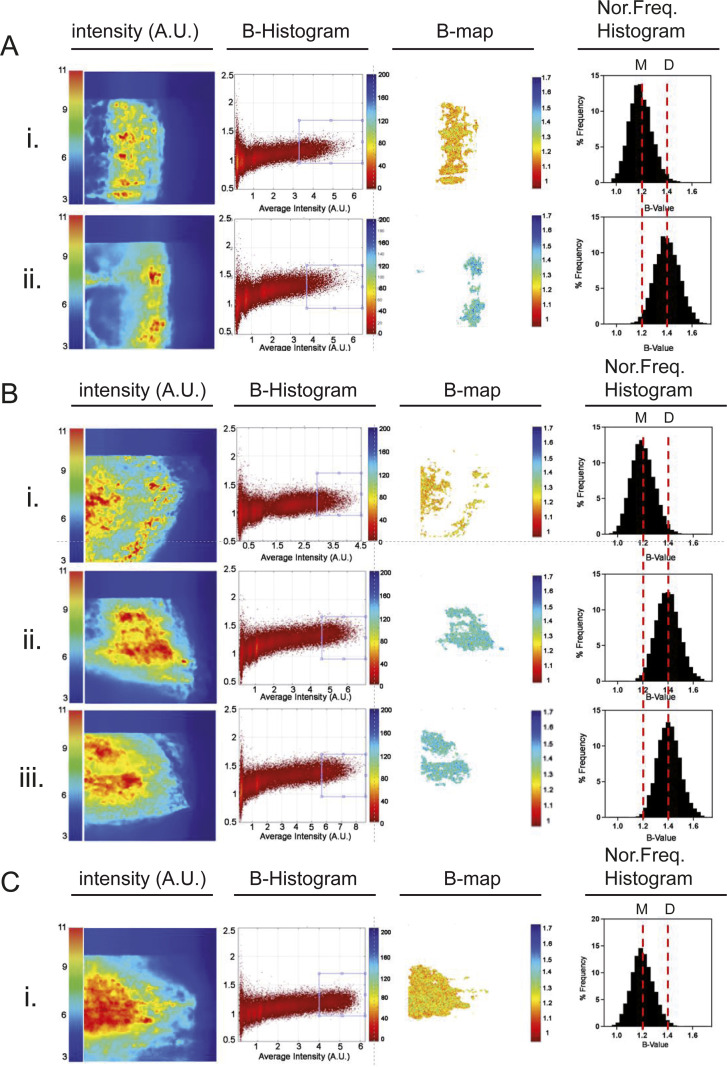
Representative examples of single-cell N&B analysis steps. **(A)** Representative analysis is shown for each of the conditions below: (A) cells expressing dim-IRE1 at low levels either (i) untreated (n = 20 cells) or (ii) treated with 10 nM AP20187 (n = 22 cells). **(B)** Cells expressing dim-IRE1 at high levels either (i) untreated (n = 23 cells), or (ii) treated with 10 nM AP20187 (n = 25 cells), or (iii) treated with 50 nM AP20187 (n = 30 cells). **(C)** Cells expressing dim-IRE1 at high levels treated with Tm (n = 23 cells). From left to right: the intensity of the time stack images is averaged and the B-histogram obtained as described in the Materials and Methods section. The blue rectangle in the B-histogram shows the region of interest, above the background threshold, which is used to obtain the B-map. B-map values are finally represented as normalized frequency histograms (Norm. Freq. Histogram). The red lines mark the B-central values (average brightness). A shift of the red line is observed upon induced dimerization (Zamai et al, 2019b). M = Fv2E-IRE1-mEGFP monomers, D = Fv2E-IRE1-mEGFP dimers.

Secondly, we resorted to Number & Brightness (N&B; Zamai et al, 2019a), a moment analysis capable of measuring the average number of molecules and their oligomerization state (variance of the fluorescence brightness) in each pixel from a series of TIRF microscopy images (Fig S3A–C). To do this, we took advantage of the fact that all our mutants were tagged with mEGFP (see Fig 2A) and we tested whether upon AP20187 treatment, dim-IRE1 was indeed present as a dimer, or instead it could form oligomers. We performed the analysis in conditions of low and high expression of dim-IRE1 and in the presence of two different concentrations of AP20187. N&B data clearly show that in basal conditions, dim-IRE1 is present as a monomer, both at low and at high concentrations. Treatment with AP20187 does lead to the formation of dim-IRE1 dimers (Figs 4B and S3A and B). However, under no conditions we were able to detect oligomers of dim-IRE1, not even at high levels of expression of dim-IRE1 and in the presence of AP20187 in saturating amounts.

In essence, our data support the idea that dim-IRE1 dimers are not capable of self-phosphorylation and do not become endonuclease-competent. However, a dimer can phosphorylate other dimers in trans, provided that they reach a sufficiently high local concentration. Reaching the activation threshold might entail active recruiting systems. Thus, the ability of IRE1α to form oligomers or big clusters would be key to facilitate this process. We speculate that this indeed could be part of the mechanism that defends cells from spontaneous activation on IRE1α′s endonuclease. How this is achieved is still not clear, but the group of Mariappan has shown that the Sec61 translocon (in particular through the Sec63 subunit) can regulate the oligomerization state of IRE1 and hence its activity. This was shown to be particularly relevant in the case of prolonged activation of IRE1 signaling (Sundaram et al, 2017, 2018; Li et al, 2020).

In fact, it has been shown that excessive activation of IRE1 can lead to cell death via RIDD, a mechanism in which IRE1 endonuclease activity is unleashed against mRNAs other than XBP1. It is still obscure though whether RIDD is executed by dimers of IRE1 (Tam et al, 2014). To probe this aspect, we assessed the effect of dim-IRE1 activation on cell vitality using the crystal violet assay (Fig 4D). The prolonged expression of dim-IRE1 per se had no effect on cell vitality, not even when the dimerizable protein was expressed at very high levels. Similarly, inducing the dimerization of dim-IRE1 had no impact on cell growth, when the protein was present in low amounts. In these conditions, dim-IRE1 is fully dimerized but not phosphorylated and not active (see Figs 3A and B and 4C). Increasing dim-IRE1 expression to the levels when it has moderate endonuclease activity as can be judged by XBP1 spicing (mid-high), did not lead to cell death (Fig 4C and D) which implies that RIDD activity is not the main task of IRE1 dimer. Only when dim-IRE1 was dimerized and present in very high amounts, that is, sufficient to trigger its phosphorylation and cause efficient XBP1 splicing (Fig 4C), did we observe a clear reduction in cell vitality (Fig 4D).

In our hands, therefore, prolonged activity of IRE1 dimers seems to have a toxic effect on cells, possibly through RIDD (Fig 4C and D). One possible mechanism, which requires further investigation, would be that XBP1 is the favorite substrate of IRE1 and only upon depletion of XBP1 IRE1 switches to a pro-apoptotic function via RIDD as was suggested before (van Anken et al, 2014a).

### Phosphorylation stabilizes IRE1α dimers/oligomers in an RNase-competent conformation

**Figure 5.**
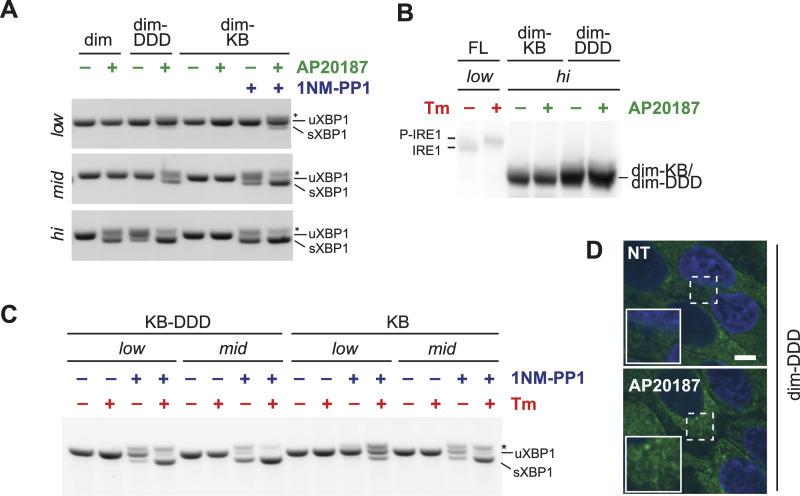
Role of nucleotide binding in endonuclease activity of IRE1. **(A)** Endonuclease activity of dimerizable IRE1α mutants at different expression levels (low, medium, high) after treatment with or without dimerizing drug (AP20187, green) and with or without 1NM-PP1 (blue). **(B)** Cells expressing FL-, dim-KB-, and dim-DDD-IRE1, at low or high levels as indicated, were treated with Tm (red) or AP20187 (green). The phosphorylation state of IRE1 mutants was assessed by Phos-tag gels and immunoblot with anti-IRE1 antibodies. dim-DDD (because of the substitutions of the three serines) and dim-KB (because of the inability to bind ATP and, hence, phosphotransfer) cannot be phosphorylated even if highly expressed. **(C)** Endonuclease activity of nucleotide binding requiring (KB-)IRE1α mutants, expressed at low and medium levels, after treatment with Tm (red) and/or NM-PP1 (blue), as indicated. KB-DDD-IRE1α was used to evaluate the possible role of phosphorylation in stabilization of the nucleotide-bound state. XBP1 splicing by both KB mutants depends on nucleotide activation (compare, for instance, lanes 1–2 with 3–4), intracellular concentration (compare lanes 3 with 7 or 4 with 8), and phosphorylation (compare lanes 3 with 11 or 4 with 12). **(D)** Confocal images of cells expressing GFP-tagged dim-DDD-IRE1. Even at high expression levels the mutant does not form foci upon treatment with AP20187. Scale bar = 10 μm.

We reasoned that if IRE1α needs to be highly concentrated, or in a higher than dimeric state to be phosphorylated, and, in turn, phosphorylation is required for RNase activity, then a phosphomimetic version of dim-IRE1α (i.e., with the three serines in the activation loop mutated to aspartic acid (dim-S724D/S726D/S729D, in short dim-DDD-IRE1α)) would be capable of splicing XBP1 upon the addition of the dimerizing agent, also at lower expression levels. This was indeed the case: dim-DDD-IRE1α was able to restore XBP1 splicing, only in the presence of AP20187 and—as expected—at lower concentrations than dim-IRE1α (Fig 5A). Remarkably, the phosphorylated/phosphomimetic dimer retained full endonuclease activity even if it did not form foci (Fig 5D). In our hands, formation of stable big oligomers was not required to splice XBP1, but phosphorylation was.

### Nucleotide binding provides an additional layer of regulation of IRE1α activity

It has been previously shown that the kinase-dead IRE1α mutant I642G can be rescued by the addition of a nucleotide analog 1NM-PP1 that allosterically activates its RNase domain, mimicking adenosine nucleotide binding (Papa et al, 2003; Han et al, 2009). By the addition of 1NM-PP1, therefore, it is possible to bypass the kinase step and activate dim-IRE1α endonuclease activity irrespective of its phosphorylation state.

**Figure S4.**
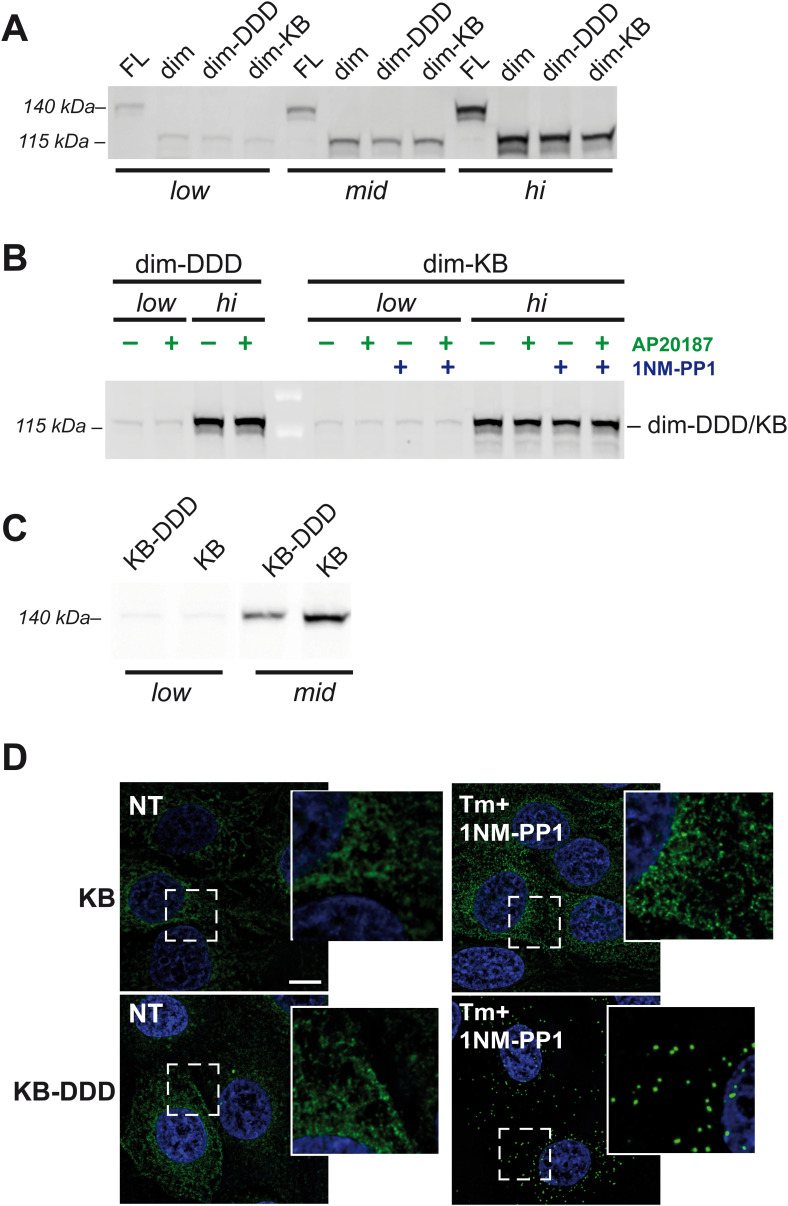
Expression levels of dimerizable and full-length kinase bypass IRE1 mutants. Phosphorylation facilitates formation of IRE1 foci. **(A)** Cells reconstituted with FL-, dim-, dim-DDD- and dim-KB-IRE1 were induced with different concentrations of doxycycline to induce comparable low, medium, and high expression levels, as indicated. IRE1 mutant proteins were revealed by immunoblot with anti-IRE1 antibodies. **(B)** Cells expressing dim-DDD- and dim-KB-IRE1 at low or high levels were treated with AP20187 (green) and/or 1NM-PP1 (blue) as indicated. Immunoblot analysis with anti-IRE1 antibodies shows that none of the treatments alter the expression levels of either of the two IRE1 mutants. **(C)** Similar to Fig S1C–E, HeLa cell lines expressing KB- and KB-DDD-IRE1α mutants under the TetON-inducible promoter were treated with different concentrations of doxycycline to adjust the expression level of the transgenes to comparable levels between each other (low and medium levels in this case). Aliquots from the cell lysates were resolved electrophoretically and the blots decorated with anti-IRE1α. **(D)** Microscopy images of cells expressing GFP-tagged KB- and KB-DDD-IRE1 at the medium expression level (see (C)). The boxed area in each image is shown magnified on the right, to facilitate the comparison. KB-DDD-IRE1 forms distinct foci upon treatment with Tm and 1NM-PP1, whereas KB-IRE1 does not. All images have been acquired with the same magnification; scale bar (valid for all panels) = 10 μm.

Taking advantage of this, we engineered the I642G mutation within the dim-IRE1α, generating a kinase bypass (dim-KB-IRE1) mutant that allows independent control of both dimerization and RNase activation steps. As expected, dim-KB-IRE1α displayed no phosphorylation, as revealed by Phos-tag gel (Fig 5B), and similar to AP20187, treatment with 1NM-PP1 did not alter the expression level of the dim-KB mutant (Fig S4B). However, the mutant was perfectly able to restore XBP1 splicing when expressed at the medium expression level in the presence of the dimerizing drug and 1NM-PP1 (Fig 5A). Structural studies of the yeast Ire1p suggested that nucleotide binding promotes oligomerization (Lee et al, 2008). Thus, the role of phosphorylation could be that of stabilizing the most efficient ADP-bound state of the protein.

Sharing an identical ER luminal domain, it is unlikely that dim-IRE1α, dim-DDD, and dim-KB-IRE1α differ in their affinity for the dimerizing drug. Thus, if phosphorylation and subsequent formation of a stable nucleotide-bound state would happen within a dimer, all three dimerizable mutants should be activated at similar expression levels (Fig S4A). However, dim-IRE1α was activated at significantly higher expression levels than dim-DDD and dim-KB (Fig 5A), implying that to be phosphorylated (and consequently ADP-bound) higher order oligomers have to be formed, even if perhaps transiently.

Dimerization remained a key step, regardless of phosphorylation. Indeed, in the absence of the dimerizing drug, the phosphomimetic mutant dim-DDD-IRE1 was not fully active even when expressed at high levels (Fig 5A). Its low activity in this condition might be due to spontaneous formation of dimers of the phosphomimetic mutant even if it is still impeded by the absence of the luminal domain. In contrast, dim-IRE1α requires the presence of the dimerizing drug even at very high expression levels. Taken together, these observations indicate that phosphorylation contributes to the stability of dimeric or higher ordered structures of IRE1α, as previously suggested by structural studies (Korennykh et al, 2009) and recombinant human IRE1 kinase/endoribonuclease proteins (Le Thomas et al, 2021). The same may be true for nucleotide binding: as shown in Fig 5A, the dim-KB mutant is more prone than dim-IRE1α to display RNase activity—it is active at medium and high expression levels upon 1NM-PP1 binding even in the absence of the dimerizer. These findings suggest that nucleotide binding stabilizes IRE1α dimers and/or oligomers, which can eventually trigger RNase activity. Phosphorylation can also contribute to the stability of the nucleotide-bound state.

We further confirmed it using full-length IRE1α mutants in which kinase activity can be bypassed (KB, mutation I642G) and with the triple phosphomimetic mutation at S724D/S726D/S729D. Upon stress, phosphomimetic kinase bypass IRE1 (KB-DDD) was active in the presence of 1NM-PP1 at low expression levels (Figs 5C and S4C), whereas KB-IRE1 needed higher expression levels. This result confirmed that phosphorylation is important for IRE1α activity and that it may favor formation and/or stability of higher ordered structures of IRE1α that are more efficient in XBP1 cleavage.

This hypothesis is supported by the fact that upon treatment with Tm and 1NM-PP1, KB-DDD-IRE1 readily formed foci (Fig S4D), in contrast to KB-IRE1. Even if we cannot formally exclude that KB-DDD-IRE1α has a higher affinity for 1NM-PP1 than KB-IRE1, these data indicate that IRE1 phosphorylation favors cluster formation. It was also shown before that it is not binding of the nucleotide analog per se that is necessary for clustering of IRE1, but ATP hydrolysis and/or the accompanying conformational change (Ricci et al, 2019).

Thus, dimerization–oligomerization, phosphorylation, and nucleotide binding can organize a circle of mutual support, which altogether promotes endonuclease activity of IRE1. Recruitment at exit sites (as in case of ATF6 [Schindler & Schekman, 2009]) might favor encounters between IRE1α dimers, and possibly screening IRE1 from deactivating phosphatases. Negative regulation would be instead exerted by binding to BiP, either directly or mediated by Sec61/Sec63 (Bertolotti et al, 2000; Sundaram et al, 2017; Li et al, 2020), and/or other players that may limit IRE1a congregation.

## Discussion

The main finding of our study is that dimerization is not sufficient to trigger IRE1α phosphorylation. This key step in IRE1α activation must proceed through the formation of higher order oligomers. According to protein–protein docking and molecular dynamics simulations, tetramers represent the most favorable configuration that IRE1α molecules can adopt (Carlesso et al, 2020). This requirement might be an important way to prevent the generation of unwanted stress signals. It seems therefore that cells need to congregate a sufficient number of IRE1α molecules in a restricted area to activate XBP1 splicing. Instead, “diluting” IRE1α would lead to decreased activity of the stress sensor. Accordingly, it is of note that IRE1β suppresses endonuclease activity of IRE1α probably by forming heterocomplexes (Grey et al, 2020).

**Figure 6.**
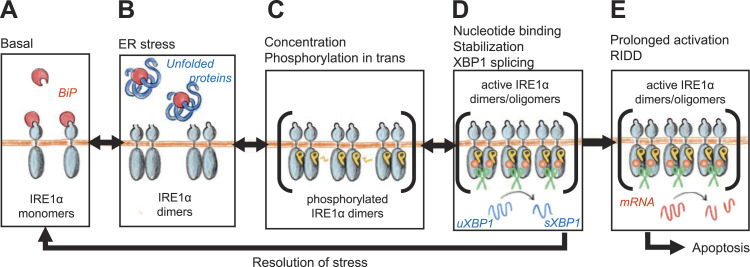
Steps of IRE1α activation. Schematic representation of the sequential steps that lead to full-blown IRE1α activation (see the text for a more detailed description). **(A)** Under the basal unstressed condition, IRE1 is bound to BiP and mostly monomeric; (B) accumulation of unfolded proteins in the ER lumen and disengagement of BiP allow the formation of IRE1 dimers; (C) collisions between dimers enable transphosphorylation. Although dimers are few or sparse, activation is mild and might be transient. Clustering and formation of signaling foci would boost dramatically IRE1 phosphorylation by increasing the local concentration of dimers, segregating them away from phosphatases. **(D)** Phosphorylation favors stability of higher order oligomers and nucleotide binding. This in turn enhances endonuclease activity, which is exerted first on XBP1 mRNA, with pro-survival effects. **(E)** Upon prolonged activation, IRE1 endonuclease activity diverts toward other mRNAs (RIDD) with pro-apoptotic effects.

The following chain of events can be hence reconstructed for IRE1α activation (Fig 6A–E):

(A) In basal conditions, free BiP is available. The binding to BiP keeps IRE1 in a monomeric state.

(B) When unfolded proteins accumulate, BiP is titrated away from IRE1. IRE1 can form dimers (Bertolotti et al, 2000; Pincus et al, 2010). Alternatively, IRE1 forms inactive dimers at the steady state (Belyy et al, 2022).

(C) Encounters between IRE1α dimers allow transphosphorylation. These encounters would be very rare events unless the IRE1 local concentration is high enough. Formation of clusters (foci) is instrumental to that, favoring and maintaining phosphorylation, possibly screening phosphorylated dimers from phosphatases.

(D) IRE1α phosphorylation triggers conformational changes that favor stability of higher order oligomers (Ricci et al, 2019) and nucleotide binding (Lee et al, 2008). This in turn enhances endonuclease activity, which is exerted first on XBP1 mRNA, with pro-survival effects.

(E) If endonuclease activity goes on for too long and XBP1 mRNA runs out, IRE1 then commits to RIDD, splicing other mRNAs and switching to a pro-apoptotic function.

When ER stress is low, few dimers are formed and even fewer are able to meet and be cross-phosphorylated. At the submaximal level of activation, IRE1α might sustain some XBP1 splicing, but dephosphorylation could prevail. When the intensity and/or duration of stress increases, IRE1α may form clusters to gain maximal phosphorylation and enzymatic activity. Allowing protection from phosphatase, formation of supramolecular complexes can further increase/prolong IRE1α activation, eventually driving cells into apoptosis. However, cluster formation is not necessary for XBP1 splicing.

Through the mechanism we propose, cells may adapt the response to the intensity of stress, limiting excessive RNase activity. IRE1α clusters would be part of the extreme measures that cells undertake when stress is overwhelming. Tilting from pro-survival to pro-apoptotic programs, this mechanism would be key in stress-related diseases and a pharmacological target.

## Materials and Methods

### Plasmids and IRE1α transgenes

Plasmids for lentiviral transduction of the IRE1α mutant and chimeric transgenes were derived from pTETTAB in which the transgene is placed under the TetTight-inducible promoter (Cohen et al, 2017).

The three IRE1α serines undergoing phosphorylation (Prischi et al, 2014) were mutated into either alanine or aspartic acid using reverse oligos GGTACCCCtGcACGtCtcgcGAAAgcaTGCCTGCCCACTGCCAGCTT and GGTACCCCatcgCGaCGatcGAAAtcaTGaCgGCCCACTGCCAGCTTCTTGC generating respectively S724A/S726A/S729A (AAA-IRE1) and S724D/S726D/S729D (DDD-IRE1).

Dimerizable IRE1α (dim-IRE1) was constructed by replacing the luminal domain of murine IRE1α-GFP (amino acids 1–349), or the respective IRE1α variant, with the Fv2E domain as it was done before for another ER stress sensor, Perk (Lu et al, 2004; Lin et al, 2009). The interface mutant of IRE1α (IM-IRE1), with disrupted dimerization surface, was generated by K121Y substitution according to Li et al (2010). Finally, kinase bypass IRE1α that can be activated allosterically by 1NM-PP1 (KB-IRE1) was obtained by I642G mutation, as described previously in Papa et al (2003); Han et al (2009).

GPI-mEGFP and GPI-mEGFP-mEGFP constructs used for N&B calibration were described previously (Hellriegel et al, 2011).

### Cell lines

Clones expressing the different IRE1α mutants at the desired levels were obtained by reconstituted HeLa-µs IRE1 KO (clone μ910-6) as described previously (Bakunts et al, 2017; Cohen et al, 2017). In brief, these cells contain a cassette for the expression of murine secretory Ig-μ chains under the mifepristone-inducible promoter (Sirin & Park, 2003; Bakunts et al, 2017), and they no longer express endogenous IRE1α because of CRISPR-mediated inactivation of the gene. Reconstitution was achieved by lentiviral transduction with the plasmids described above (Cohen et al, 2017). All IRE1α mutant genes are placed under the TetON tight promoter, which allows the inducible and tunable expression of the protein upon the addition of doxycycline.

Cell lines generated for this study (HeLa-µs IRE1α S724A/S726A/S729A [AAA-IRE1], HeLa-µs IRE1α K121Y [IM], HeLa-µs Fv2E-IRE1α [dim-IRE1], HeLa-µs Fv2E-IRE1α S724D/S726D/S729D [dim-DDD-IRE1] and HeLa-µs Fv2E-IRE1α I642G [dim-KB-IRE1], HeLa-µs IRE1α N906A [RD-IRE1], HeLa-µs IRE1α I642G [KB-IRE1], HeLa-µs IRE1α I642G S724D/S726D/S729D [KB-DDD-IRE1]) are summarized in Table S1.

Table S1 List of cell lines.

All cell lines in this study were ultimately derived from HeLa S3 cells, of which the genotype was confirmed by PCR single-locus technology (Bakunts et al, 2017).

### Reagents and treatments

The expression of μs under the MifOn promoter was triggered by treatment with 0.5 nM mifepristone (Bakunts et al, 2017). The expression of IRE1α variants was generally induced with doxycycline for 48–72 h before the experiment. The dose of doxycycline to use was determined empirically for each individual cell line and adjusted in order to achieve close-to-endogenous (low), medium, or high levels of expression, as determined by comparative Western blots.

For allosteric activation of kinase bypass IRE1 mutants (KB-, KB-DDD- and dim-KB-IRE1), 1NM-PP1 (MedChemExpress) was used at the concentration of 7 μM. Dimerization of Fv2E domains was induced by treatment with AP20187 (MedChemExpress), at a concentration of 10 nM, unless stated otherwise.

Kifunensine (Sigma-Merck) was used at the concentration of 30 μM.

Rabbit anti-human IRE1α was from Cell Signaling (3,294), and rabbit-α-human phosphoIRE1α (S724) was from Abcam (ab48187).

The crystal violet assay was performed as previously described (Bakunts et al, 2017).

### Analysis of IRE1α phosphorylation and activity

To detect IRE1α phosphorylation, we used the method previously described in Yang et al (2010). Cells were lysed in 50 mM Tris–HCl, 150 mM NaCl, 60 mM octyl glucoside, pH 7.4, containing phosphatase inhibitor cocktails 2 and 3 (Sigma-Aldrich). Proteins were separated by SDS–PAGE on a 5% polyacrylamide gel containing 20 μM Phos-tag (NARD, Wako Chemicals), performed at 15 mA for 2–2.5 h. The gel was transferred to nitrocellulose (Bio-Rad) and decorated with anti-IRE1α antibodies.

IRE1α RNase activity was determined by the XBP1 splicing assay, as described previously (Bakunts et al, 2017).

### Fluorescence microscopy

Light microscopic images were acquired with an UltraView spinning disk confocal microscope operated by Volocity software (PerkinElmer) or a DeltaVision Ultra microscope (GE Healthcare) with oil immersion objective at 100x (Olympus 100X/1.45, 1-UXB240) magnification, and deconvolved with instrument software.

### TIRF microscopy

Dim-IRE1 in HeLa cells was imaged by TIRF microscopy, taking advantage of the evanescent illumination (Poulter et al, 2015). We used a Leica AM TIRF microscope equipped with an iXon 897 EMCCD camera (Andor^TM^ Technology) and a thermostatic chamber for maintaining 5% CO_2_ atmosphere at 37 ± 0.5°C (Meyer Instruments). Dim-IRE1 cells were serum-starved overnight and imaged 2 h after incubation with the dimerizing drug AP20187. For N&B studies, the camera was calibrated as previously described (Unruh & Gratton, 2008). Time stacks of images (700–1,000 frames, 256 × 256 pixels, 124 nm/pixels) were collected with a TIRF field of 250 nm, at 488 nm excitation and 2 ms/frame (24 ms accumulate cycle time). Photobleaching and photodamaging because of repeated illumination were avoided by choosing a different cell in a different field of view at each replicate time-point. We also prevented distortion of the brightness because of intensity changes during acquisition using a total acquisition time within 11–22 s. Moreover, we discarded time series showing fluorescence intensity changes >5% (Trullo et al, 2013). Cells with the high overexpression of dim-IRE1 were not analyzed.

### N&B data analysis

N&B is a pixel-by-pixel moment analysis (Dalal et al, 2008). It measures the average number of molecules (n) and the average brightness (ε) in each pixel, which are related to the average fluorescence intensity (〈I〉) as follows:

〈I〉=ε n (1)

When a protein labeled with an mEGFP of brightness 1xε associates as a homodimer, the complex will carry 2 mEGFP labels, and the N&B analysis will yield a molecular brightness of 2xε. Populations of mixed oligomers will have a mean brightness 〈ε〉 weighted by the fractional intensity (fi = Ii/ΣnIn) of the individual “n” components: 〈ε〉 = Σn fn εn (Hellriegel et al, 2011).

At low fluorescence intensity (low expression levels of fluorophores), the brightness (ε) of the fluorophore can be derived from the fluorescence fluctuation amplitudes caused by the diffusion of fluorescent molecules in and out of the observation volume. Thus, the average brightness 〈ε〉, expressed in [(counts/molecule) × dwell time], is obtained by computing 〈B〉 (apparent pixel brightness), the ratio of the variance to the average intensity (σ^2^/〈I〉), at each pixel from the equation:

〈B〉=1+〈ε〉 (2)

〈B〉 is normalized as follows:

$$\text{average}\;\text{brightness}\;\text{ratio}=\left(\langle B_{i}\rangle \;‐\;1\right)/\left(\langle B_{0}\rangle \;‐\;1\right)=\langle \varepsilon_{i}\rangle /\langle \varepsilon_{0}\rangle$$ (3)

where 〈B_i_〉 is the average B-value measured at time t_i_ after the addition of AP20187, and 〈B_0_〉 is the average B-value measured at the time t_0_ = 0 (before AP20187 addition), which, in all cases, agreed with the value of the monomeric GPI-mEGFP (monomeric reference, 〈B_c_〉 = 〈B_0_〉 = 1.2 and 〈ε_c_〉 = 〈ε_0_〉 = 0.2). As a control, GPI-mEGFP and GPI-mEGFP-mEGFP, in HeLa cells, were images as monomer and dimer brightness references, respectively. The experimental procedure was previously described in Hellriegel et al (2011); Zamai et al (2019a). Briefly, TIRF time stacks were averaged to compute the pixel-by-pixel averaged intensity (〈I〉). The apparent pixel brightness (B) was obtained from the ratio between the variance and the average intensity at each pixel as B = σ^2^/〈I〉. B versus 〈I〉 values were mapped as B-histograms. For the analysis, a region of interest was selected above the background. The brightness of each pixel of the region of interest was computed as a B-value and is shown in the B-map. Finally, the analysis of B-value distributions was used to determine the central value, representing the average apparent B-value of the image. The central value was calculated as the SD among the brightness values of the independent pixels divided by the square root of the number of pixels. At least 1,200 pixels (i.e., 1,200 brightness values) were analyzed in each single cell using GraphPad (GraphPad Software Inc.) (Zamai et al, 2019a, 2019b).

## Supplementary Material

Reviewer comments
